# Noninvasive Technique for the Diagnosis of Patent Ductus Arteriosus in Premature Infants by Analyzing Pulse Wave Phases on Photoplethysmography Signals Measured in the Right Hand and the Left Foot

**DOI:** 10.1371/journal.pone.0098763

**Published:** 2014-06-03

**Authors:** Sabrina Goudjil, Fatiha Imestouren, Aurelie Armougon, Lucie Razafimanantsoa, Mahdi Mahmoudzadeh, Fabrice Wallois, André Leke, Guy Kongolo

**Affiliations:** 1 Neonatal Intensive Care Unit, Amiens University Hospital, Amiens, France; 2 Pediatric Cardiology, Amiens University Hospital, Amiens, France; 3 INSERM U1105, GRAMFC, Jules Verne University of Picardie, Amiens, France; Scuola Superiore Sant'Anna, Italy

## Abstract

**Objective:**

To evaluate the impact of patent ductus arteriosus (PDA) on the pulse phase difference (PPD) between the left foot (postductal region) and the right hand (preductal region).

**Materials and Methods:**

PPD was determined from arterial photoplethysmography signals (pulse waves) measured by infrared sensors routinely used for pulse oximetry in 56 premature infants less than 32 weeks gestation. Only infants with significant PDA (sPDA) diagnosed by echocardiography were treated with ibuprofen (for 3 days). Patients were classified according to whether or not they responded (Success/Failure) to this treatment. The Control group was composed of infants in whom ductus had already closed spontaneously at the time of the first echocardiography. The 3 groups were compared in terms of PPD at the beginning (T1) and at the end (T2) of the study. For patients in the Failure (n = 17) and Success groups (n = 18), T1 corresponded to the first day of treatment and T2 to the day after completion of the course of ibuprofen. In the Control group (n = 21), T1 corresponded to 1 to 3 days of life (DOL), and T2 to 4–6 DOL.

**Results:**

Compared to the Control group, PPD was higher in the Failure (at T1 and T2) and Success (at T1) groups characterized by sPDA. After ibuprofen therapy, PPD in the Success group decreased to about the level observed in the Control group. The area under the ROC curve of PPD for the diagnosis of sPDA was 0.98 (95% CI 0.96–1); for an optimal cut-off of PPD ≥1.65 deg/cm, the sensitivity was 94.2% and the specificity was 98.3%.

**Conclusion:**

In this study, PPD was correlated with ductus arteriosus status evaluated by echocardiography, indicating involvement of the ductal shunt in the mechanism of redistribution in systemic vascular territories. PPD can be considered for the diagnosis of hemodynamically significant PDA.

## Introduction

Patent ductus arteriosus is common in premature infants born before 32 weeks and is associated with high morbidity (i.e., pulmonary hemorrhage, necrotizing enterocolitis, acute renal failure, intraventricular hemorrhage, etc.) and mortality, especially in neonates with a birthweight less than 1000 g [1], [2]. This condition is characterized by a ductal shunt that diverts part of the aortic blood flow towards the pulmonary circulation, resulting in pulmonary vascular overload and decreased blood flow to systemic organs. Recent data suggest that the decrease in systemic blood flow is more pronounced in postductal regions than in preductal regions [3]. Treatment with ibuprofen or indomethacin is prescribed only in infants with significant patent ductus arteriosus and surgical ligation is proposed in the case of failure or contraindication to medical treatment. The characteristics of arterial pulse wave propagation are dependent on the mechanical properties of the blood vessel (inner diameter and wall thickness) and blood viscosity, according to the Moens-Korteweg law [4]. The fact that local hemodynamic conditions have an influence on blood vessel mechanics suggests that, in the presence of significant patent ductus arteriosus, pulse wave propagation properties would probably be impacted to varying degrees on preductal and postductal vessels [5], [6].

Some authors consider that blood oxygen saturation is low in congenital heart diseases and suggest to perform screening in newborn by criteria of postductal arterial oxygen saturation <95% or a difference > 3% between pre and postductal saturation [7], [8].

The present study, using photoplethysmography signals recorded by conventional pulse oximetry technology measured the arterial pulse wave phase [9] in the right hand (preductal vascular area) and left foot (postductal vascular area) and then calculated the difference between these two values, corresponding to the pulse phase difference (PPD).

The objective of this study was to evaluate the impact of PDA on the PPD and to analyze PPD as a marker of ductal shunt severity (as classified by echocardiographic criteria).

## Materials and Methods

This pilot study was conducted during the first week of life in premature infants, at the time of diagnosis and evaluation of patent ductus arteriosus in the Amiens University Hospital neonatal intensive care unit between August 2010 and July 2013.

### Ethics Statement

The study protocol was approved by the Amiens University Medical Center Institutional Review Board (CPP Nord Ouest II-France, IDRCB: 2008-A00704-51). Infants between 26 and 32 weeks of gestational age investigated by echocardiography for the diagnosis of ductus arteriosus were eligible to participate in the study after obtaining written informed consent from their parents. The Institutional Review Board approved this consent procedure.

### Study protocol

The study started at the time of echocardiographic assessment of ductus arteriosus status (T1). Patients with significant PDA were treated with a 3-day course of ibuprofen. A second echocardiography was performed on the day after completion of ibuprofen therapy to confirm ductus closure (T2). Photoplethysmography signals were recorded at T1 and T2 in the right hand and left foot, corresponding to preductal and postductal regions, respectively. Patients treated by catecholamine infusion were not included in this study. Clinical characteristics of the patients are summarized in Table 1.

**Table 1:** Clinical characteristics of the study population.

	Failure group (n = 17)	Success group (n = 18)	Control group (n = 21)	p
**GA, median (IQR), wks**	27 (26.7–29)	28.9 (27–30)	29.4 (28–31)	ns
**PNA, median (IQR), d**	2.2 (1.2–2.9)	2.4 (1.6–2.8)	1.9 (1.4–2.5)	ns
**BW, median (IQR), g**	1080 (870–1280)	1230 (900–1410)	1250 (1000–1440)	ns
**CRIB II, median (IQR)**	8.1 (7.8–9.7)	8.3 (4.5–9)	7.7 (4.2–10)	ns
**Hb, median (IQR)**	10.7 (9.9–13.1)	12.6 (10.9–16)	12.4 (11.8–15.5)	ns
**FiO2, median (IQR), %**	21 (21–29)	21 (21–26)	23 (23–27)	ns
**Vaginal delivery, %**	52.9	55.5	57.1	ns
**Antenatal steroids, %**	70.6	61.5	71.4	ns
**Tracheal ventilation, %**	64.7	55.5	52.4	ns
**Midazolam, %**	17.6	16.7	19.0	ns
**Sufentanyl, %**	35.3	33.3	33.3	ns

GA, gestational age; PNA, Postnatal age; BW, Birth weight; CRIB II, clinical risk index for babies score; Hb, Hemoglobin; FiO_2_, Fractional inspired concentration of oxygen; IQR, interquartile interval

### Doppler echocardiography

Echocardiography protocols and diagnostic criteria for PDA complied with those published by several authors in many specialized journals [10], [11]. Patent ductus arteriosus was diagnosed on echocardiography by visualization of a left-to-right ductal shunt between the aortic isthmus and the pulmonary artery [12], [13]. In our study, patent ductus arteriosus was considered to be significant when it was associated with at least one of the two main echocardiographic criteria of severity (ductal diameter >2 mm and/or left atrial-to-aorta diameter ratio (LA/Ao) >1.4) with at least one other criterion of severity (ductal shunt flow velocity (V_ductus_) <1.2 m/s, mean left pulmonary artery flow velocity (LPAV) >0.40 m/s and end-diastolic LPA flow velocity (EDLPAV) >0.20 m/s). In our study, echocardiographic assessments were performed by neonatologists and were systematically reviewed by a pediatric cardiologist, who was not informed of PPD finding, to validate the results.

### Photoplethysmography signals acquisition and extraction from the bedside monitor

**Figure 1:**
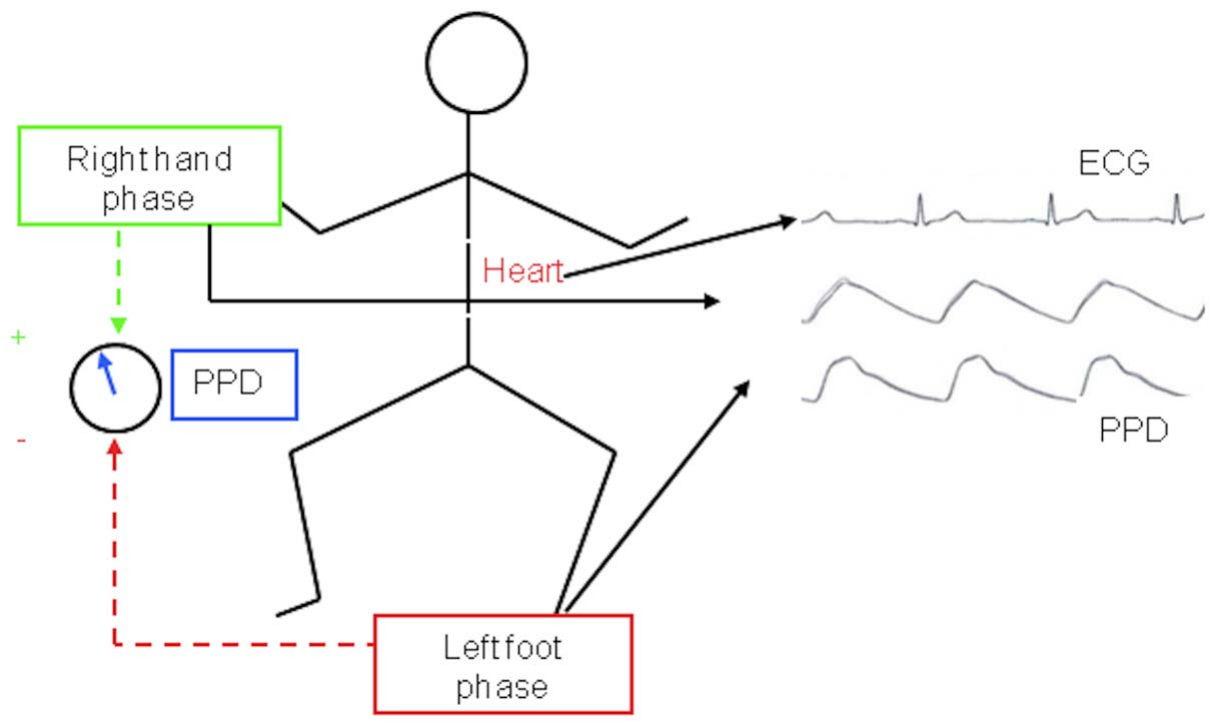
Photoplethysmography signals acquisition technique using infrared sensors. Acquired signals were used to compute pulse phases corresponding to preductal (right hand sensor) and postductal (foot sensor) regions and their difference, the PPD.

Photoplethysmography signals were acquired in the right hand (preductal territory) and left foot (postductal territory) (Figure 1) using pulse oximetry infrared sensors [9], [14].

**Figure 2:**
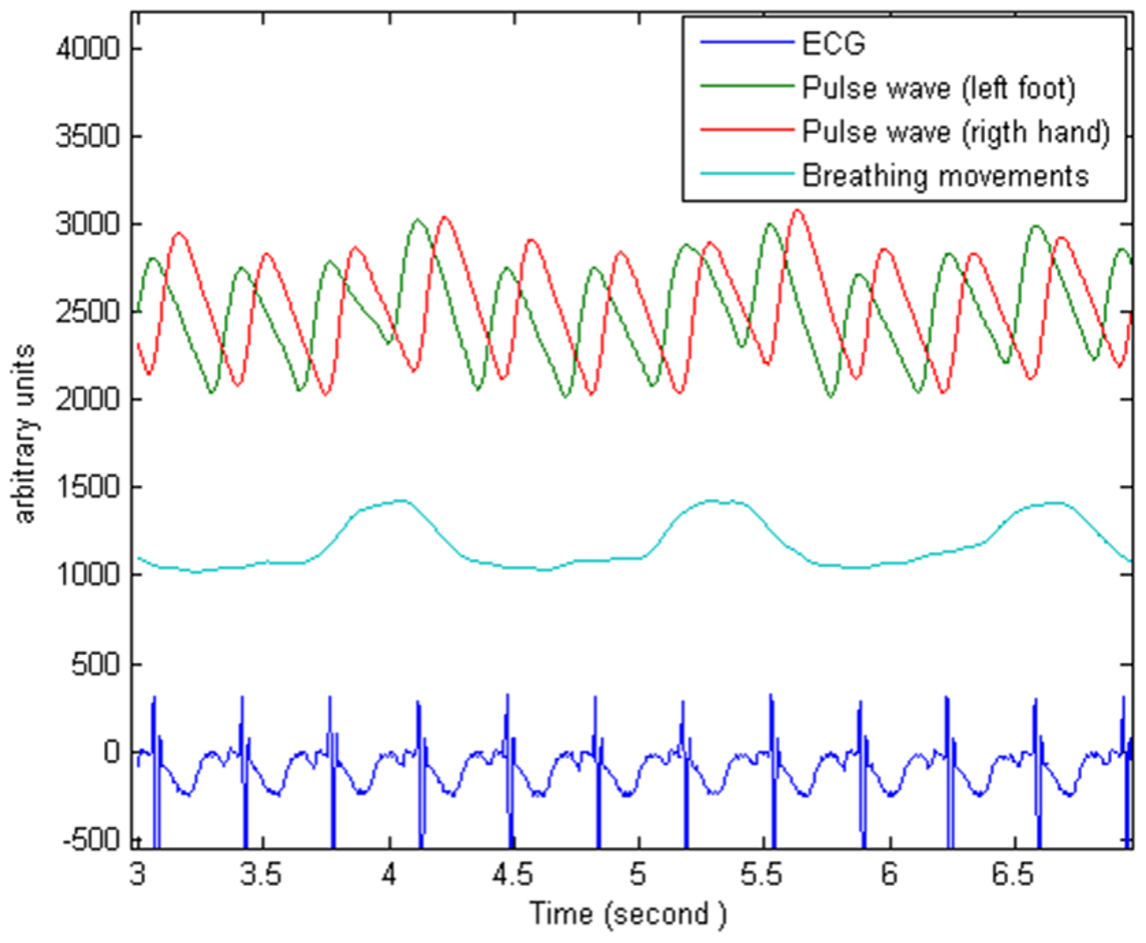
Raw photoplethysmography signals. These photoplethysmography traces show a delay between the recordings from the right hand and left foot in an infant with PDA.

Electrocardiogram and thoracic respiratory movements were also recorded at the same time and were then extracted from the bedside monitor (MP40, Philips) to a laptop for further processing (Figure 2). The photoplethysmography signal sampling frequency was 125 Hz.

### Empirical Mode Decomposition (EMD) of photoplethysmography signals

The technique developed in this study is based on determination and comparison of instantaneous angular position - the phase - of the arterial wave pulse in the hand and foot. This operation is only possible with mono-component signals such as those obtained by applying empirical decomposition [15], [16]. EMD is an adaptive technique for non-linear and non-stationary signal analysis based on the signal characteristics. In our study, EMD decomposed the photoplethysmography signal into stationary components with a very narrow frequency band corresponding to Intrinsic Mode Functions (IMF):
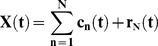
(1)


In this expression, X(t) corresponds to the original data; c_n_(t) are intrinsic mode functions (IMF) of specific time-scale obtained after sifting the original data; and r_N_(t) is the residue. In this study, extended EMD (EEMD) was applied to avoid mode mixing problems resulting from intermittent signals that can induce instability of EMD operations. In the EEMD version, many trials (n = 100) were repeated, each with a different sequence of white noise (σ = 0.2 times the amplitude of X(t)) added to the original signal X(t) before performing decomposition.

IMFc then represented the mean of a set of 100 trials. Technical and computational details on EMD and the improved EEMD version can be found in the papers by Huang *et al*. [17] and Lo *et al.* [16].

**Figure 3:**
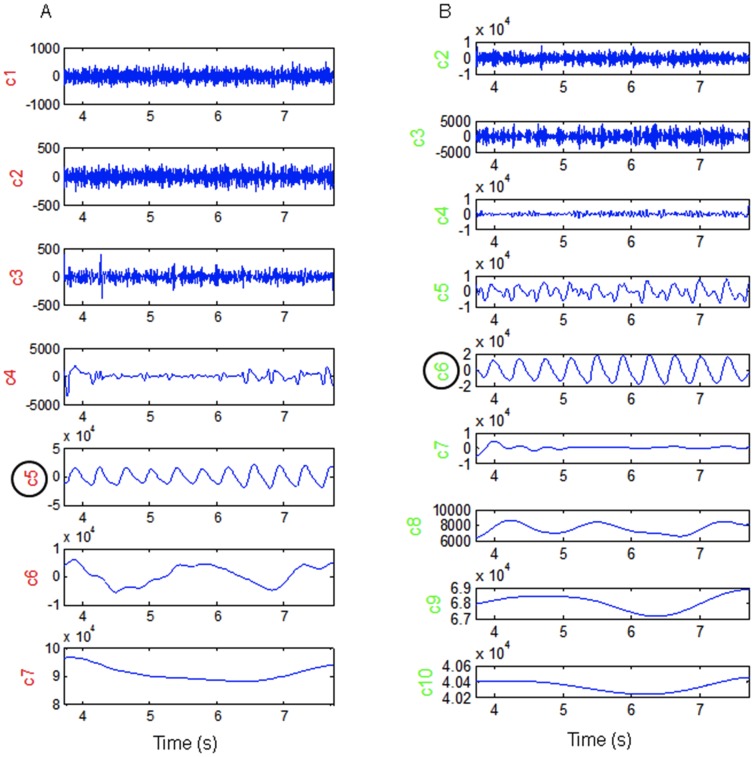
Intrinsic Mode Functions resulting from empirical decomposition of photoplethysmography signals. This figure shows the decomposition of signals recorded in the left foot (A) and right hand (B) in elementary functions (IMF) characterized by selective time-scale. This allowed the identification of components relating to cardiac activity (c5 in figure 3A and c6 in figure 3B). In these graphs, IMFs are organized in ascending order from top to bottom.

Figure 3 shows the results of decomposition of signals from the foot (A) and hand (B) after the EMD sifting operations. Each line corresponds to a distinct IMF related to physiologic phenomena with a specific time-scale.

In Figure 3, the intrinsic mode functions are organized in ascending order of time-scales from top to bottom. Depending on its complexity, a signal can be decomposed into a large number of IMF, each related to phenomena with a specific time-scale. In this study, the EMD features allowed identification of components (IMFc) whose energy was mostly concentrated around the time-scale corresponding to the patient's cardiac activity, which also allowed elimination of all global trends due to respiratory cycles, and artifacts from active body movements and other experimental errors.

### Instantaneous phase of photoplethysmography signals

The IMFc was derived from decomposition of the signals recorded in the foot and hand. The IMFc was then submitted to Hilbert transformation [17] to obtain the amplitude and instantaneous phase of their analytic signals. 
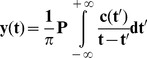
(2)where P indicates the Cauchy principal value, and y(t) the result of Hilbert transformation of the IMF signal c(t).

The analytic signal can be expressed as:
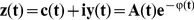
(3)where A(t) is the instantaneous amplitude,
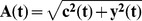
(4)and φ(t), the instantaneous phase:
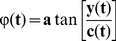
(5)


**Figure 4:**
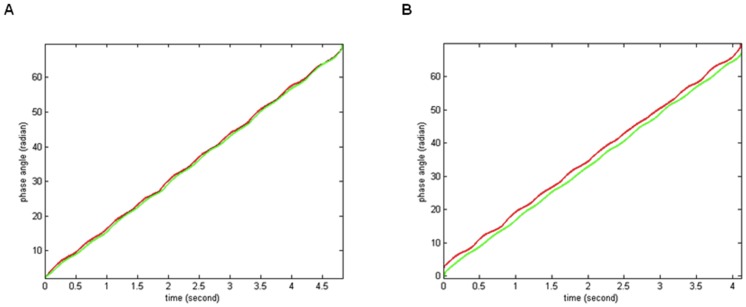
Unwrapped phase obtained by applying Hilbert transform to cardiac IMF. The phase curves obtained from an infant in the Control group (A) are very close, while those recorded in an infant from the Failure group are more distinct (B).

Figure 4 shows the curves of instantaneous unwrapped phases of cardiac component (IMFc) in pulse signals from the hand (red) and foot (green). The graph on the left (Figure 4A) shows two very close phase curves corresponding to recordings from an infant in the Control group with closed ductus; the graph on the right (Figure 4B) shows a more marked difference between the two phase curves that were recorded in an infant of the Failure group with significant patent ductus arteriosus.

### Determination of the pulse wave phase difference (PPD)

**Figure 5:**
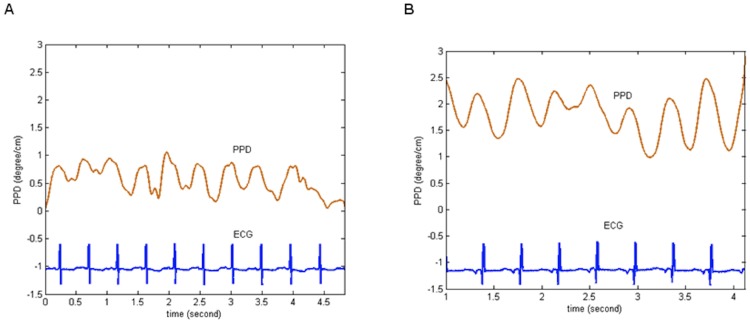
Time-course of PPD (deg/cm) normalized with respect to body height. (A) Infant with closed ductus; (B) Infant with patent ductus arteriosus. ECG, electrocardiogram; PPD, the pulse phase difference.

The pulse wave phase difference (PPD) was obtained by subtracting the phase of the signals recorded in the right hand from that recorded in the left foot (Figure 5).

**Figure 6:**
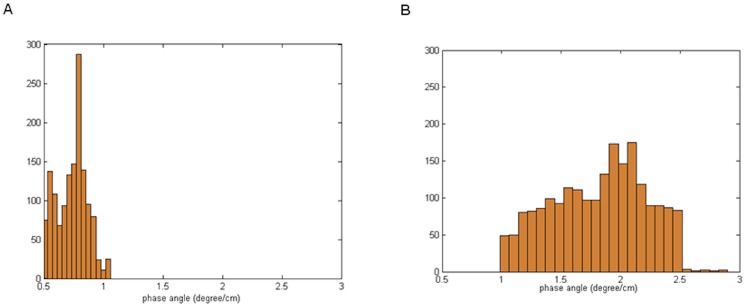
Histogram of distribution of PPD values (deg/cm). (A) Infant with closed ductus: the histogram of PPD is narrow and concentrated to segment less than 1 degree/cm; (B) Infant with patent ductus arteriosus: the histogram of PPD is broad, with values above 1 degree/cm.

PPD can also be analyzed on a simple statistical histogram (Figure 6) or on a Rose diagram angular graph.

In this study, PPD was determined 3 times for each investigation on randomly selected segments of photoplethysmography signals during quiet periods with regular electrocardiogram complexes and breathing movements. The three estimations were applied to compute the mean value of PPD for further analyses and were also used for determination of the coefficient of variation to confirm the reproducibility of the PPD technique [18].

### Patient classification

All patients with PDA were treated by intravenous ibuprofen according to the usual treatment protocols used in our neonatal unit (*Pedea*, 3 days of intravenous treatment: 10 mg/kg on the first day, 5 mg/kg on the 2^nd^ and 3^rd^ days). Echocardiography was repeated after ibuprofen therapy to confirm PDA closure.

Patients in whom ductus arteriosus was closed or constricted after ibuprofen therapy were classified in the Success group and those with persistent significant PDA despite medical treatment were classified in the Failure group.

A Control group was composed of infants with spontaneous closure of the ductus since the first echocardiographic assessment at 1–3 days and at the follow-up assessment performed at 4–6 days of life. Patients of Control group did not receive any treatment.

### Statistical analysis

Quantitative variables were expressed as the median (IQR, 25^th^–75^th^ percentile) and qualitative variables were expressed as number (percentage). Nonparametric Kruskal-Wallis ANOVA was performed to compare PPD between the three groups (Failure, Success and Control) and the Wilcoxon test was used for comparison of paired observations between the first (T1) and second (T2) evaluation time-points. Statistical significance was defined as p<0.05.

## Results

### Clinical characteristics of the study population and management

A total of 56 premature infants were included in the study. Thirty five infants presented significant PDA diagnosed by echocardiography and were treated by ibuprofen. Following the 3-day intravenous ibuprofen therapy, constriction or closure of the ductus arteriosus was observed on echocardiography in 18 of these infants (6F/12M) (Success group). Seventeen patients (5F/12M) still had significant PDA after ibuprofen therapy (Failure group).

The clinical characteristics of the 21 infants (10F/11M) in the Control group, and those in Success and Failure groups are summarized in Table 1.

### Echocardiographic characteristics of ductus arteriosus

Echocardiographic characteristics are summarized in Table 2. Ductus diameter was >2 mm in the Success and Failure groups at T1. At T2, ductus diameter in the Success group had decreased to very low values (about 0.5 mm).

**Table 2:** Echocardiographic characteristics of the study population, median (IQR).

Group (n)	Failure (n = 17)		Success (n = 18)		Control (n = 21)	
	T1	T2	T1	T2	T1	T2
**Ductal diameter (mm)**	2.5 (2.3–2.5)	2.6 (1.8–2.9)	2.4 (2.2–2.6)	0.55 (0–0.9)**‡	-	-
**V. ductus (m/s)**	1.0 (0.8–1.9)	1.4 (1.0–2.4)	1.3 (1.1–2.3)	2.6 (2.1–2.9)*‡	-	-
**LPAV (m/s)**	0.67 (0.5–0.8)	0.77 (0.7–0.9)	0.55 (0.5–0.7)	0.3 (0.3–0.51)	0.3 (0.2–0.5)	0.38 (0.3–0.5)
**EDLPAV (m/s)**	0.3 (0.2–0.35)	0.22 (0.1–0.4)	0.25 (0.2–0.4)	0.1 (0.0–0.3)*	0.03 (0–0.04)	0.02 (0–0.05)
**LA/Ao**	1.42 (1.3–1.8)	1.36 (1.2–1.7)	1.4 (1.3–1.6)	1.2 (1.1–1.3)*	1.3 (1.1–1.4)	1.3 (1.2–1.4)

(**‡**): Data only for patients with constrictive ductus; **V. ductus**, Maximal ductal flow velocity; **LPAV**, Mean left pulmonary artery flow velocity; **EDLPAV**, End diastolic left pulmonary artery flow velocity; **LA/Ao**, Left atria-to-Aorta diameter ratio; **IQR**, Interquartile interval; **T1**, the day of diagnosis by echocardiography; **T2**, the day after the end of treatment with ibuprofen. For patients in the Control group, T1: 1–3 day of life and T2: 4–6 days of life; paired comparison T1 vs T2 in the same infants, (*): p<0.05, (**): p<0.01.

As expected in the SUCCESS group at T2, ductal flow velocity became very high (2.6 m/s) and end-diastolic LPA velocity became lower than the values measured when the ductus was fully patent (i.e, in the Failure group at T1 and T2, and in the Success group at T1).

### Pulse wave phase difference (PPD) between the right hand and the left foot

In this study, the coefficient of variation of PPD was 11.6% for all measurements.

**Figure 7:**
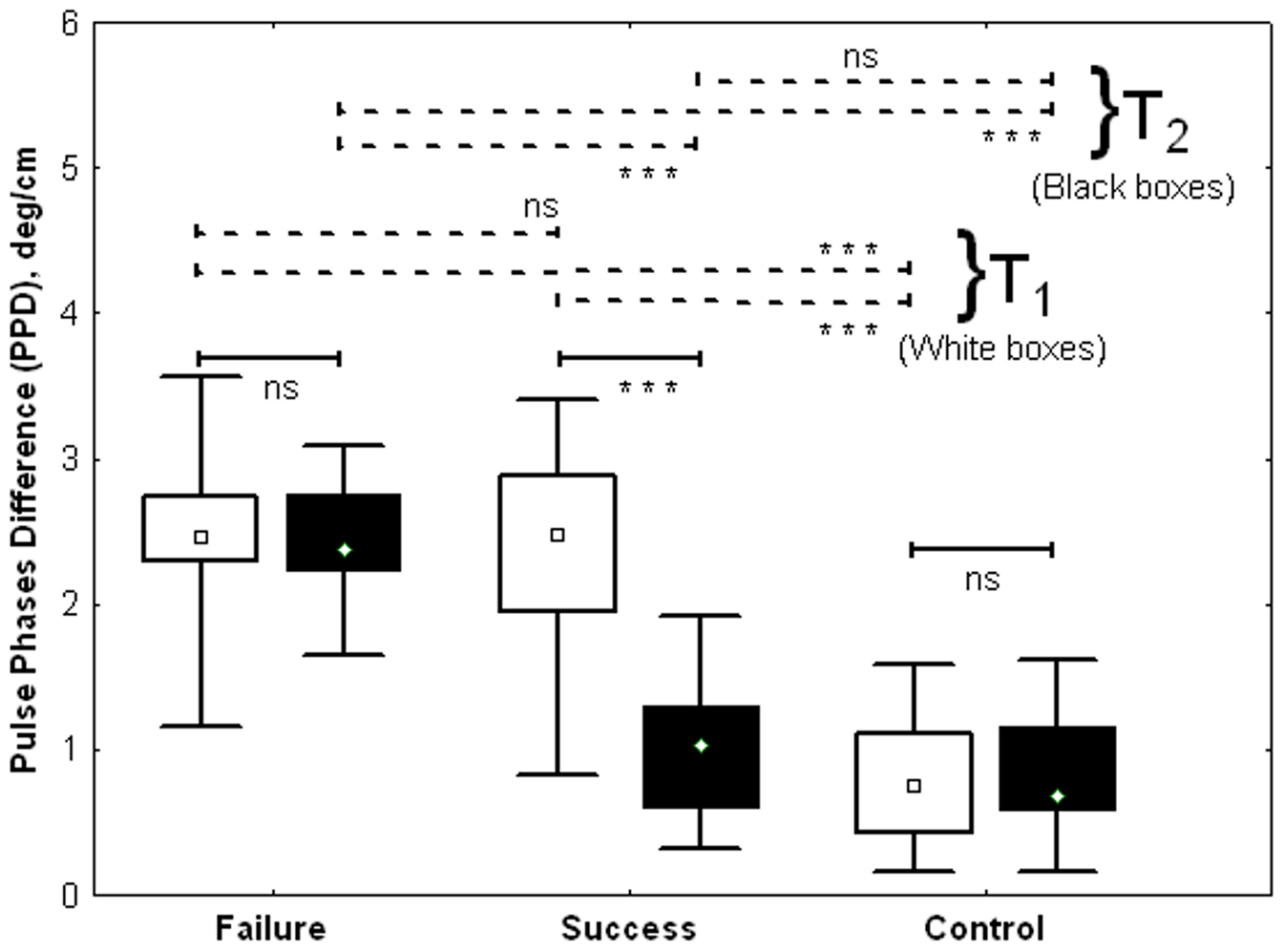
PPD during the study in function of classification in Failure, Success and Control groups (Median; Box: 25th -75th percentile; Whisker: Minimum-Maximum). The values of PPD (deg/cm) at the beginning of the study at T1 are represented with white boxes and at the end of the study (T2), with black boxes (***: p<0.001; ns: non significant).

PPD results for each group are summarized in Figure 7.

The 3 groups were not homogeneous in terms of PPD at T1 (Kruskal-Wallis, H(2, N = 56) = 35.6; p<0.001. The PPD value in the Control group (T1: median, IQR: 0.74 (0.44–1.12)) was lower than those observed in the Failure group (T1: median, IQR: 2.46, 2.30–2.75; p<0.001) and in the Success group (T1: median, IQR: 2.48, 1.95–2.89; p<0.001). PPD was not significantly different between the Failure and Success groups.

The 3 groups also differed in terms of PPD at T2 (Kruskal-Wallis, H (2, N = 56) = 35.3; p<0.001). PPD decreased significantly between T1 and T2 in the Success group (T2: median, IQR: 1.03, 0.61–1.30; p<0.001, Wilcoxon) and became similar to the PPD values observed in the Control group (T2: median, IQR: 0.68, 0.59–1.16; p = 0.19). PPD did not change between T1 and T2 in the Failure group (T2: median, IQR: 2.38, 2.24–2.76; p = 0.24, Wilcoxon). The PPD value at T2 in the Failure group was higher than that measured in the Success (p<0.001) and Control groups (p<0.001). PPD did not change between T1 and T2 in the Control group (p = 0.77).

**Figure 8:**
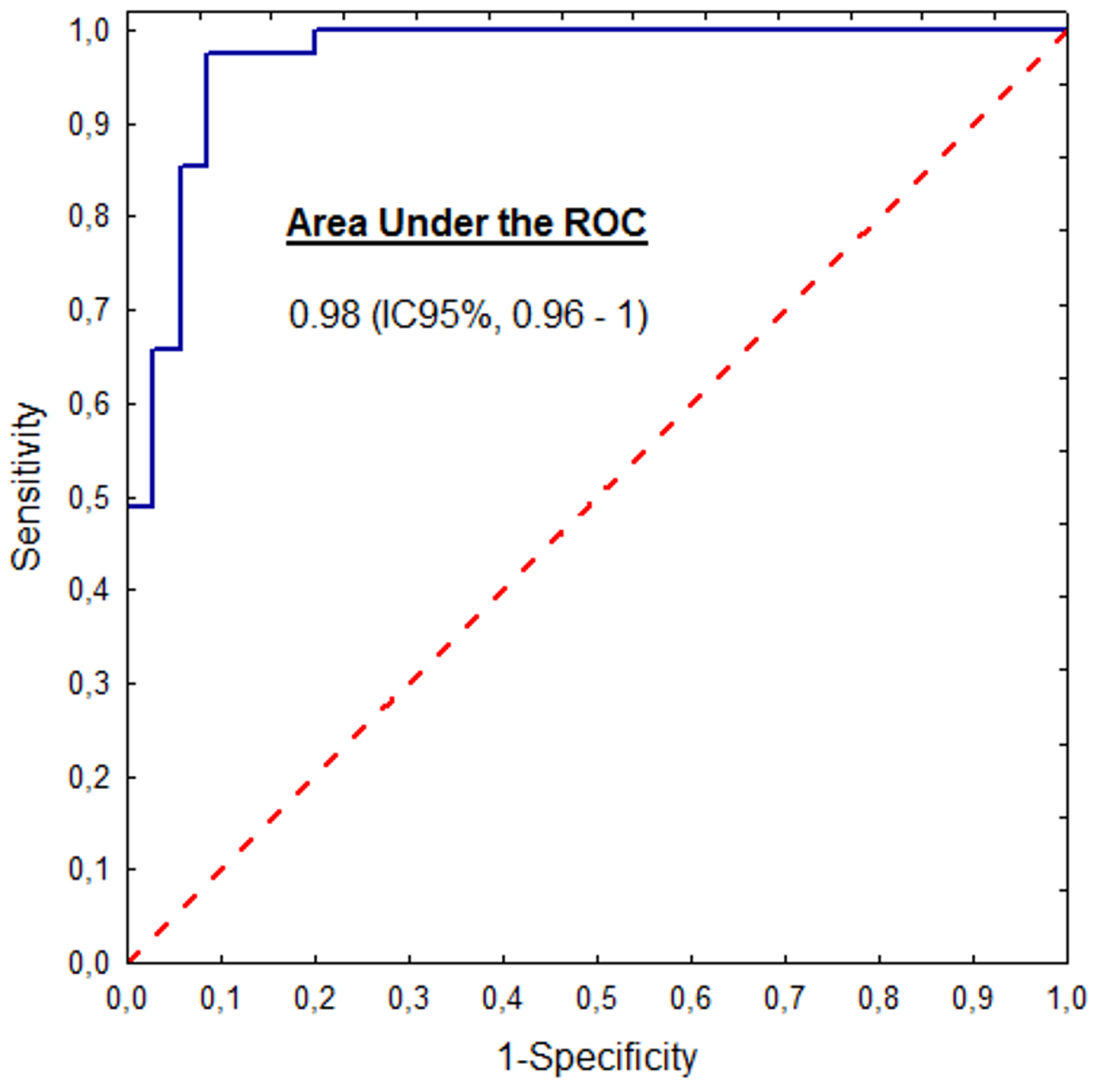
ROC curve of PPD for discrimination of infants with or without significant patent ductus arteriosus. For the optimal cut-off of PPD ≥1.65 deg/cm, the area under ROC curve was 0.98 (0.96–1) with a sensitivity of 94.2% and a specificity of 98.3%.

Analysis of the PPD technique for the diagnosis of hemodynamically significant patent ductus arteriosus showed an optimal PPD cut-off of ≥1.65 deg/cm, with an area under the ROC curve of 0.98 (95% confidence interval, 0.96–1), a sensitivity of 94.2% and a specificity of 98.3% (Figure 8).

## Discussion

The primary objective of this study was to analyze the pulse wave phase difference (PPD) between the preductal (right hand) and postductal (left foot) regions as function of the significant or closed-constricted status of the ductus arteriosus in preterm infants.

### PPD and ductus arteriosus status

The main findings from this study are that closed ductus is associated with low PPD values and significant patent ductus arteriosus is associated with high PPD values.

This is corroborated by the lower PPD values observed in patients of the Control and Success group at T2 after ibuprofen therapy. In these patients, the closed status of the ductus was confirmed by echocardiography. Moreover, in the Success group, ductus status changed from open to closed between the start and end of treatment while PPD decreased from high (about 2.4 deg/cm) to low values (about 1 deg/cm).

The highest PPD values were measured in patients with significant patent ductus arteriosus, corresponding to patients of the Success group at the start of the study (T1), and patients of the Failure group at both T1 and T2. As the study groups mainly differed in terms of closure of the ductus and not in terms of any other of the characteristics analyzed (Table 1), these results suggest an impact of ductus status on PPD. To our knowledge, such data have not been previously reported in the literature.

### Other potential determinants of PPD

The pharmacologic effects of ibuprofen are less likely to be associated with the PPD differences observed between patients in the Control group and patients with PDA, as, at time-point T2, PPD was very high in Failure group but low in the Success group; however, patients in both groups received ibuprofen therapy.

In the Moens-Korteweg equation [19], wave velocity is dependent on blood viscosity, blood vessel diameter, wall thickness and wall elasticity: 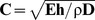 (C  =  pulse velocity, D  =  vessel diameter, E  =  wall elasticity, h  =  wall thickness and ρ  =  blood viscosity).

Other factors such as mean blood flow in the vessel and mean blood pressure also influence pulse wave velocity, but via indirect mechanisms [20].

PDA is known to alter the distribution of blood flow to various organs, causing pulmonary vascular overload and decreased blood flow in the systemic circulation [1], [21]. Redistribution disorders are also observed in the various regions of the systemic circulation [22], [23]. The blood volume supplying the brain and upper limbs would be relatively preserved [5], as it is derived from large arteries arising from the aortic arch proximal to the ductus arteriosus [24]. Therefore, as vessel diameter is proportional to mean blood flow in a given territory [25], artery diameter would remain unchanged in preductal regions and would be significantly decreased only in postductal regions due to a more marked reduction of perfusion in postductal regions due to the ductal shunt [25].

Several other factors may also affect the elastic properties of vessels [26]: as catecholamine infusion can alter vascular tone [27], patients treated with catecholamines were therefore excluded from this study.

Blood viscosity influences arterial pulse propagation properties [20]. No significant change in hemoglobin was observed between the first (T1) and second examinations (T2) in any of the patients of this study, suggesting that blood viscosity remained unchanged. Moreover, no patient received blood transfusions during the study period. Some potential determinants of the PPD were recognized starting from speculation on the equation of Moens-Korteweg. However this model was validated on adults, it has been only few applications in neonates [28].

The pulse wave propagation time to a given territory also depends on the distance traveled from the heart. For this reason, in our study, PPD was normalized to the patient's body height prior to interpretation [29].

We believe that modulation of the autonomic nervous system (ANS) is also a determinant of pulse wave propagation properties due to its influence on vascular tone [30], [31].

In the Success group (responder patients), PPD decreased after closure of the ductus, but remained slightly higher (but not significantly) than the PPD value observed in the Control group (Figure 7). This aspect of PPD dynamics during treatment may suggest a memory process reflecting either a local phenomenon involving blood vessel wall mechanisms (histological, humoral.) or probably the influence of autonomic nervous system adaptations, which sometimes take many hours or days to completely resolve. Further appropriate studies are necessary to explore these hypotheses.

### Technical specificities

In our study, we used a sampling frequency of 125 Hz for the acquisition of plethysmographic signal. The determination of the sampling frequency must meet the criterion of Shannon which states that its value must be at least twice the highest frequency of its spectrum [32], [33]. Studies have shown that the upper limit of the pulse plethysmographic signal spectrum remains below 25 Hz [34], [35]. In these conditions, the sampling frequency of 125 Hz performed in our study was adequate for the plethysmographic pulse signal.

Pulse wave phase characteristics are not usually analyzed for the purposes of PDA diagnosis and this analysis requires certain mathematical and engineering skills. This technique must therefore be automated before it can be used in routine clinical practice. The PPD method requires strict symmetry of the acquisition channels used to record the two photoplethysmography signals (hand and foot), since the main property of interest in this technique corresponds to the pulse wave phase difference. If this requirement is not met, it could lead to bias in PPD results. In the present study, this problem was avoided by using 2 symmetric modules in the MP40 monitor (Philips) originally designed to measure oxygen saturation by pulse oximetry from 2 different anatomic sites.

In our study the minimum value of PPD measured between the right hand and the left foot was 0.2 deg/cm; this was observed in the control group, in infants in whom the ductus arteriosus was closed. The manufacturer of MP40 monitor (Philips) ensures that recordings are symmetrical and synchronous when the two pulse oximetry modules (Philips M3001A and Philips M1020B # A01) used are original, as in our study [36].

The PPD technique developed in our study presents a number of advantages over conventional methods based on pulse transit time, as in the study by Oishi *et al.* [37]. In the present study, the EMD algorithm used to calculate PPD operates in the phase domain taking into account nonlinearities and nonstationarities usually contained in the photoplethysmography signals [38]. Based on permanent photoplethysmography waves, it was then possible to continuously estimate PPD in contrast with the conventional pulse transit time technique, which can provide only 1 estimation per cardiac cycle, dependent on R-peak detection on electrocardiogram (event-related approach) [39], [40].

### Study limitations

PPD values are difficult to interpret due to the absence of reference values from large populations of healthy preterm infants. In this pilot study, the reproducibility of PPD was only estimated by the coefficient of variation (CV = 11.6%). In fact, when introducing a new measurement's system, reproducibility should be evaluated in terms of inter/intra operator and inter/intra session variability. This precaution was not respected in our study because it was difficult to impose repeated examinations (cardiac echography.) to these tiny premature infants hospitalized in the NICU, as they were in very critical clinical state.

In future studies, efforts should be undertaken to provide a more complete analysis of the variability associated with the PPD technique.

Photoplethysmography signals were recorded only from the right hand and left foot. The decision to place a pulse oximetry sensor at the right hand was justified by its location in the arterial preductal territory. For compliance with the study protocol and to minimize the uncontrollable sources of variations, the left foot was chosen as the postductal position in all infants. However, measurements of the postductal position could be the right foot.

In future studies, it would be interesting to verify the sensitivity of this technique when measurement sites are changed from right to left hand since the origin of the left subclavian artery from the aortic arch is also proximal to the ductus arteriosus [3], [24].

The echocardiography technique consists of detection of ultrasound energies reflected on measured structures enabling measurement of distances and flow velocities used to determine the severity of patient ductus arteriosus. In contrast, PPD is based on the temporal properties of pulse wave propagation and could therefore provide original information on PDA hemodynamics, independent of the parameters measured by echocardiography.

### The post-hoc analysis of the statistical power

This pilot study provided the first quantitative data on the interest of PPD in the diagnosis of patent ductus arteriosus. Hence, it became possible to perform a post-hoc analysis of the statistical power for the PPD technique.

The design of our study was an anova model with within-effects (repeated measurements of PPD before and after ibuprofen treatment) and between-effects (the 3 groups: Failure, Success, Control) and within-between interactions.

When we considered that each of the 3 groups comprised only 17 patients (size of the smallest group), the statistical power was >80% for the within, the between and the within-between effects.

Using equal size groups is recommended to avoid overestimating the statistical power when in actually, the anova study involves unequal groups [41]. Moreover, this is a requirement in the GPower3 program used in this study for calculation of the statistical power [42]. The results of this analysis (statistical power >80%) suggest that a total population of 56 infants should be adequate for this study.

## Conclusion

PPD is a new noninvasive approach for the evaluation of PDA which proceeds in the analysis of photoplethysmography waves recorded in the right hand and the left foot using conventional pulse oximetry sensors. In this study, PPD measurements demonstrated good correlations with ductus status and were very reproducible: PPD was higher in patients with significant patent ductus arteriosus than in those with restricted or closed ductus. PPD can therefore be considered in addition to echocardiography which is the reference technique for diagnosis and evaluation of the ductus arteriosus. Automation of the PPD technique is required before considering its use in routine clinical practice.
